# Increased Psychological Distress during COVID-19 and Quarantine in Ireland: A National Survey

**DOI:** 10.3390/jcm9113481

**Published:** 2020-10-28

**Authors:** Tom Burke, Anna Berry, Laura K. Taylor, Owen Stafford, Eddie Murphy, Mark Shevlin, Louise McHugh, Alan Carr

**Affiliations:** 1 School of Psychology, University College Dublin, D04 F6X4 Dublin, Ireland; annanibheara@gmail.com (A.B.); laura.taylor@ucd.ie (L.K.T.); stafford.owen@yahoo.co.uk (O.S.); eddie.murphy2@hse.ie (E.M.); louise.mchugh@ucd.ie (L.M.); alan.carr@ucd.ie (A.C.); 2 Health Service Executive, CHO 8 (Laois/Offaly), R34 YFW6 Laois, Ireland; 3 School of Psychology, Queen’s University Belfast, Belfast BT7 1NN, UK; 4 Department of Psychology, University of Ulster (Psychology), Coleraine BT52 1SA, UK; m.shevlin@ulster.ac.uk

**Keywords:** psychological distress, public, quarantine, COVID-19, mental health, Ireland

## Abstract

Background: The emergence of the coronavirus pneumonia (COVID-19) resulted in a global pandemic. The psychological impact of an epidemic is multifaceted and acute, with long-term consequences. Methods: A cross-sectional online survey-based design was employed, assessing the psychological impact of COVID-19 on members of the Irish public during the quarantine period of COVID-19 in Ireland. Participants were invited to complete the Depression, Anxiety, and Stress Scale-21 (DASS-21) retrospectively (prior to quarantine) and during the quarantine period, as well as measures of illness perceptions, well-being, and a bespoke measure (the Effects of COVID Questionnaire, ECQ), which assessed perceptions of COVID-related stresses associated with personal concerns, caring for children, caring for aging parents, as well as gratitude. Results: A total of *n* = 1620 entered the survey platform, with a total of *n* = 847 surveys completed by members of the Irish public. Entry into COVID-19 quarantine was associated with significant increases in clinically significant symptoms of depression, stress, and anxiety. The ECQ reliably assessed a range of COVID-19-related stresses and had large and significant correlations with the DASS-21. Conclusions: The COVID-19 quarantine was associated with stresses and significant increases in symptoms of depression, anxiety, and stress in a national Irish cohort. The public require increased access to mental health services to meet this increase in COVID-19-related psychological distress.

## 1. Introduction

December 2019 saw the emergence of a novel severe acute respiratory syndrome, coronavirus pneumonia (COVID-19) outbreak, which subsequently became a global pandemic [1]. COVID-19 elicits challenging psychological and psychiatric responses due to its sudden and unpredictable nature, creating a sense of uncertainty and vulnerability, while challenging individuals’ sense of personal and societal safety [2]. This may be amplified by the treatment-resistant nature of COVID-19 to common medications and the delay in contraction to symptom onset [3]. Patients, health professionals, and the general public face increasing psychological demands and pressure which may in itself lead to challenges with anxiety, fear, depression, and sleep difficulties, all of which need to be considered and targeted in the overall deployment of the disease control measures [4].

COVID-19 is associated with a significant mental health burden both in the acute phase and the long-term from people who are exposed to the virus and those not directly exposed. Anxiety, depression, cognitive impairment, delirium, psychosis, irritability, insomnia, and post-traumatic stress disorder are prevalent following COVID-19 infection [5,6,7,8]. One-third of the first 153 COVID-19 cases in the UK were diagnosed with new-onset mental health problems including psychosis (43%), cognitive decline (26%), and affective disorder (17%) [9]. Further to the psychological response to COVID-19, in a recent COVID-19 study using a national cross-sectional survey based design in Italy [10], it was found that previous history of trauma and medical problems, or having an acquaintance infected was associated with higher levels of depression and anxiety.

With a view to reduce infection and control the outbreak of a virus, many countries undertake stepped measures such as social distancing, the reduction and cancellation of large public gatherings, self-isolation recommendations, quarantine in a dedicated facility, and mass public quarantine (Public Health England, 2020). While quarantine can be necessary during major infectious disease outbreaks from a population-health perspective, a recent systematic review suggests that quarantine itself is often associated with negative psychological and physical effects [1,11]. For some, the psychological impact of being in a pandemic is wide-ranging [11], significant, and long-lasting [12,13], requiring effective and accessible psychological support be put in place as early as possible.

In recent years, much of the research into the psychological sequalae following public quarantine has resulted from similar epidemics (e.g., Severe Acute Respiratory Syndrome (SARS) circa. 2003; Equine influenza circa. 2008; and Swine Flu circa. 2009 [H1N1 Influenza]). Research suggests that quarantine may result in a higher prevalence of symptoms of psychological distress [14], emotional disturbance [15], depression [16], stress [17], low mood with irritability and insomnia [18], post-traumatic stress symptoms [19], anger [20], and emotional exhaustion [21]. Low mood (73% of 903) and irritability (57% of 903) are among the most prevalent symptoms of psychological distress reported [18]. People quarantined because of being in close contact with those who potentially had SARS [19] reported various negative responses during the quarantine period: over 20% (230 of 1057) reported fear, 18% (187) reported nervousness, 18% (186) reported sadness, and 10% (101) reported guilt. However, not all studies have found evidence of psychological distress following quarantine. For example, [22] compared undergraduates who had been quarantined with those not quarantined immediately after the quarantine period and found no significant difference between the groups in terms of post-traumatic stress symptoms or general mental health problems. Recently, the typical responses to COVID-19 have been reported as panic, fear to go out, excessive disinfection, disappointment, fear, irritability, aggressive behavior, and extreme optimism or pessimism [23].

Mental health risks associated with COVID-19 have yet to be systematically studied; however, the emerging literature on COVID-19 as well as previous studies on infectious disease outbreaks provide insights into probable risk factors and correlates of mental health challenges and chronic psychological distress [24]. There is also emerging evidence that specific members of society, e.g., parents, may be experiencing additional psychological distress due to increased and unstable financial demands, school closures, and suspended recreational outlets, which would have support personal and familial coping [25,26]. A better classification and quantification of mental health and psychological needs following COVID-19 will allow for the appropriate consideration of therapeutic frameworks, service-based funding considerations, intervention integration through non-routine modalities, and to consider service models and accessibility for those vulnerable and in need [27].

The primary aim of the study was to investigate the mental health and well-being of adults in Ireland during the quarantine period of the COVID-19 crisis and examine the reliability and validity of a new instrument for assessing stresses and things for which people felt grateful that were specifically related to the COVID-19 crisis (the Effects of COVID-19 Questionnaire [ECQ, Berry and Carr, 2020]). For the purpose of this study, the quarantine period is defined as the period of time in which the initial national lockdown occurred in Ireland. The following specific questions were addressed:

- Were mean levels and rates of depression, anxiety, and stress significantly higher during the quarantine period?
- Did mean levels and rates of depression, anxiety, and stress differentially increase significantly for those caring for children or older aging parents during the quarantine period?
- What were the rates of stresses and things that people felt grateful for that were specifically related to the COVID-19 crisis (as assessed the ECQ)?
- What were the psychometric properties of the ECQ?
- Were the COVID-19-related stresses and gratitude assessed by the ECQ correlated with indices of mental health and well-being?

## 2. Materials and Methodology

### 2.1. Participants

A cross-sectional survey-based design was employed to recruit a public sample through the use of media outlets, social media, and professional networking websites in Ireland, during the period of mass public quarantine (effective 27 March 2020 to 8 June 2020, as per Irish government authorization). A total of *n* = 1620 entered the survey platform, with a total of *n* = 847 completing each of the survey questionnaires fully. The response sample was predominantly female, and the age range was reflected between 18 and 76 years of age. Further details on the participant results are below. Inclusion criteria were being over the age of 18; living in Ireland at the time of quarantine; and participants were required to read an information sheet and provide consent before proceeding to the questionnaire. Participants were excluded if they did not meet the inclusion criteria.

### 2.2. Materials

All information provided was self-reported and completed online through the use of *Qualtrics* (SPA, London, England). Demographic information regarding gender, age, marital status, household and family composition, and years of education were collected. The following scales were used: the Depression, Anxiety, and Stress Scale (DASS-21) [28], the Warwick–Edinburgh Mental Well-Being Scale (WEMWBS) [29], the Brief Illness Perception Questionnaire (BIPQ) [30], and the ECQ (Berry and Carr 2020).

The DASS-21, which yields scores for depression, anxiety, and stress, was the primary measure for this study. Each scale contains 7 items. The Depression scale assesses dysphoria, hopelessness, devaluation of life, self-deprecation, lack of interest/involvement, anhedonia, and inertia. The Anxiety scale assesses autonomic arousal, skeletal muscle effects, situational anxiety, and subjective experience of anxious affect. The stress scale is sensitive to levels of chronic non-specific arousal. It assesses difficulty relaxing, nervous arousal, and being easily upset/agitated, irritable/over-reactive, and impatient. Scores for depression, anxiety and stress are calculated by summing the scores for the relevant items per scale; then, the DASS-21 subscale total is multiplied by 2 to give the final score for categorization into Normal, Mild, Moderate, Severe, or Extremely Severe. The reliability of the DASS-21 was considered acceptable [31] and has “good” Cronbach’s alpha values of 0.81 and 0.89 for the depression and anxiety subscales, respectively. The alpha value for the stress subscale was observed at 0.78, which is considered “fair” [31].

The WEMWBS is a 14-item measure that focuses on the positive aspects of mental health and well-being including optimism, autonomy, agency, curiosity, clarity of thought, positive relationships, positive affect confidence, and having energy to spare [29]. High scores indicate greater well-being. The reliability of the WEMWBS is noted to be “good” within a student sample, with an observed Cronbach’s alpha of 0.89 [31].

The BIPQ assesses the cognitive and emotional representations of illness. For this survey, we included the “Cognitive Perceptions” subscale adapted for COVID-19, which asks about the effect of COVID-19 on life (item 1); perceived duration of COVID-19 (item 2); control over COVID-19 (item 3); beliefs about the effectiveness of treatment for COVID-19 (item 4); and experience of COVID-19 symptoms (item 5). We further employed a single item to capture understanding of COVID-19 (item 7); items 1–5 are summed to give a total score for the “Cognitive Perceptions” scale. High BIPQ scores reflect negative perceptions of COVID-19. The reliability of the BIPQ has been shown to have a “good” Cronbach’s alpha value of 0.85 [31].

The ECQ is a bespoke 34-item tool that measures perceptions of COVID-related stresses as well as gratitude arising from the COVID-19 crisis, as developed by the second and senior author following a review of the literature and discussions as part of a doctoral thesis (Supplementary Table S1). Items 1–25 are about COVID-19-related stresses. Items 26–34 are about things participants felt grateful for arising from the COVID-19 crisis. For COVID-19-related stresses, participants were asked, “In the past month, how much stress have you experienced as a result of the following things?” For COVID-19-related gratitude, participants were asked, “In the past month, how much has your experience of the COVID-19 crisis led you to feel grateful for the following things?” For all items, there are 5 response options: none, a little, some, quite a lot, and a great deal. The ECQ contains four a priori scales: Personal Stress (items 1–13), Parenting Stress (items 14–21), Older Aging Parent Stress (items 22–25) and Gratitude (items 26–34). Items in the Personal Stress scale cover financial hardship, difficulty getting supplies, loss of social contact, loss of routine, family conflict, conflicting media information about COVID-19, witnessing or worrying about COVID-19-related illness, hospitalization, death, and long-term effects for oneself and one’s family. Items in the Parenting Stress scale (which are only relevant to respondents with children) cover school closure, preventing children having social contact with extended family and friends, helping children observe social distancing, handwashing, cough etiquette, and worrying about their health due to the presence an underlying condition that makes them vulnerable to COVID-19-related adverse outcomes. Items in the Older Aging Parent Stress scale (which are only relevant to respondents with aging parents) cover worrying about the impact of COVID-19 on older aging parents, especially loneliness, difficulty getting supplies, risk of illness, and risk of not receiving adequate medical care. Items in the Gratitude scale cover things that the COVID-19 crisis has made one feel grateful for including personal and family health, relationships, employment, social, sports and cultural events, community, schools, children’s friendships, children’s involvement in activities, and aging parents’ health and safety. The ECQ is included in the supplemental materials.

### 2.3. Procedure

This study was approved by the host institution’s research ethics committee (HS-E-20-66-Burke). Study information was disseminated through the use of a national online media outlet, informing readers of the nature of the study and inviting interested participants to take part. Social media, and other online platforms were also engaged to support recruitment. Then, interested participants followed a link to access the study information sheet, consent, and questions via *Qualtrics*. Once participant screening questions were completed, i.e., confirmation of being in Ireland during the national quarantine period, and inclusion criteria were confirmed, consent was obtained, and participants provided demographic information. Following this, participants were requested to complete the outcome measures. Participants were invited to complete the survey during the quarantine period and also to consider retrospectively their well-being, distress, and mood prior to the quarantine period. Participants were informed of that a participation raffle would take place, and they could opt in to be entered into the draw to win one of 3 €50 vouchers. Participants were made aware that they may withdraw from the study at any time during data collection by simply exiting the browser window.

### 2.4. Statistical Analysis and ECQ Validation

For continuous variables, comparisons were undertaken using MANOVA, ANOVA, and t-tests. For categorical variables, chi-square tests were used for comparisons. Bonferroni corrections were used where multiple comparisons were made. Pearson product moment correlation coefficients were used to determine associations between variables. Internal consistency reliability of ECQ scales was assessed with Cronbach’s alpha. Exploratory factor analysis was used to assess the factor structure of the ECQ. K-means clustering was used as a non-hierarchical method to quantify the responses on the ECQ subscales, with an a priori cluster set to 5, to create a categorical stratification of the DASS-21 (Normal, Mild, Moderate, Severe, Extremely Severe). IBM Statistical Software Package for the Social Sciences (SPSS) 26.0 (IBM, Armonk, NY, USA) was used for analyses.

## 3. Results

A total of *n* = 1620 entered the survey platform, with a total of *n* = 847 surveys completed. There were no significant differences on age or gender in those who completed the survey completely and those who did not, who had entered demographic details prior to exiting. The median time taken to complete the survey was 14.93 min for those who completed the survey fully. As reported in Table 1, participants were mostly female (83%), and age ranged from 18 to 76 years. The household income, education status, and relationship status can be seen in Table 1, as well as a full demographic breakdown for participants who were: (1) people who had older aging parents (OAPs; *n* = 433); (2) parents of children under 18 years of age (Parents; *n* = 269, of whom, *n* = 33 had a child but did not have older aging parents); and (3) participants who had neither children nor an older aging parent (Neither; *n* = 145) at the time of completing the survey. Within each cohort, >50% of the respondents continued to work during the COVID-19 pandemic. Supplementary Table S2 shows the outcome data under the same stratification.

### 3.1. Did Mean Levels of Depression Anxiety and Stress Increase Significantly during the Quarantine Period?

**Figure 1:**
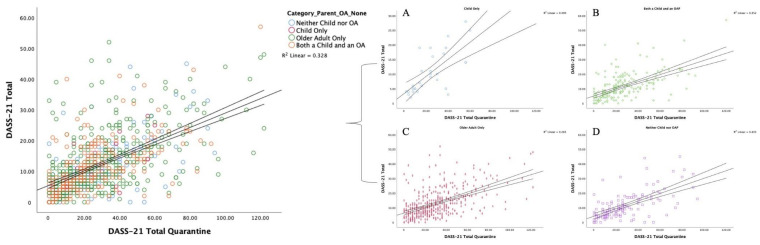
Outcomes for the summed score of the Depression, Anxiety, and Stress subscales (DASS-21) prior to, and during, the quarantine. Group stratification is illustrated as: Left: Total cohort with color demarcation; (**A**) parents with a child only i.e., no older aging parents [R^2^ = 0.490]; (**B**) both a child and an older aging parent [R^2^ = 0.352]; (**C**) older aging parents only [R^2^ = 0.283]; (**D**) neither a child nor an older aging parent [R^2^ = 0.403].

Mean DASS-21 depression, anxiety, and stress scores for the whole sample are given in the first panel of results in Table 2. When comparing the pre-quarantine outcomes to those reported during the quarantine period, there was a significant increase in mean levels of depression (pre-quarantine *M* = 7.46, quarantine *M* = 10.54, *t* = 10.50, *p* < 0.001), anxiety (pre-quarantine *M* = 5.39, quarantine *M* = 6.02, *t* = 3.39, *p* =0.001), and stress (pre-quarantine *M* = 11.99, quarantine *M* = 12.86, *t* = 2.82, *p* = 0.005). See Figure 1 for illustration.

### 3.2. Did Mean Levels of Depression Anxiety and Stress Differentially Increase Significantly for those Caring for Children or Older Aging Parents during the Quarantine Period?

Mean DASS-21 depression, anxiety, and stress scores for parents caring for children, adults caring for older aging parents, and those caring for neither type of dependant are given in the last three panels of results in Table 2. A Groups × Time MANOVA, with three groups (parent to a child, older aging parent, and neither) and two time periods (pre-quarantine and quarantine) yielded a significant Groups × Time interaction (*p* = 0.006). Post-hoc analyses show that increases in depression, anxiety, and stress from pre-quarantine to quarantine were significantly associated with having older aging parents. Post-hoc analyses with Bonferroni adjustment showed participants with older aging parents were found to have significantly elevated Anxiety pre-quarantine (*p* = 0.024) and significantly elevated Anxiety (*p* = 0.038) and Depression (*p* = 0.002) during the quarantine period, with the largest difference relative to parents with children.

### 3.3. Did Rates of Depression, Anxiety, and Stress Increase Significantly during the Quarantine Period?

Rates of mild, moderate, severe, and very severe symptoms on DASS-21 scales for the whole sample are given in the first panel of results in Table 3. When cases in mild, moderate, severe, and very severe categories were combined and compared to those in the normal category, there were increases in rates of symptoms on all three DASS-21 scales from pre-quarantine to the quarantine period. The rate of depression increased from 30 to 46.3% (*X*^2^ (1, *n* = 847) = 67.92, *p* < 0.0001). The rate of anxiety increased from 30.7 to 32.5% (*X*^2^ (1, *n* = 847) = 121.71, *p* < 0.0001). The rate of stress increased from 27.7 to 34% (*X*^2^ (2, *n* = 847) = 4.99, *p* = 0.025).

### 3.4. Did Rates of Depression, Anxiety, and Stress Differentially Increase Significantly for those Caring for Children or Older Aging Parents during the Quarantine Period?

Rates of mild, moderate, severe, and very severe symptoms on DASS-21 scales for parents caring for children, adults caring for older aging parents, and those caring for neither type of dependant are given in the last three panels of results in Table 3. To determine if there was an association between being a parent to a child, caring for an older aging parent, or having neither sort of dependent, on the one hand and symptom rates before and during quarantine on the other, 3 × 2, Group x Time, chi square tests were conducted on frequencies of symptoms outside the normal range in the three groups on both occasions.

There were significant Group × Time associations for both depression and anxiety from pre-quarantine to the quarantine period for parents caring for children, adults caring for older aging parents, and those caring for neither type of dependant (*p* < 0.001, respectively) but not specifically for stress. As reported through a detailed breakdown of the percentage of caseness and severity in Table 3, this reflects an increased severity of symptoms over time as a result of entering the quarantine period.

### 3.5. What Were the Rates of COVID-19-Related Stresses and Feelings of Gratitude?

Rates of COVID-19-related stresses and feelings of gratitude based on responses to ECQ items are given in Table 4. For each item, percentages are given for those who answered “quite a lot” or “a great deal”. Percentages are based on the number of participants for whom the items were relevant. Items 1–13 and 26–30 were relevant to all 847 participants. Items 14–21 and 31–33 were relevant to 268 parents of children. Items 22–25 and 34 were relevant to 433 people with older, aging parents.

From Table 4, it may be seen that for COVID-19-related personal stresses relevant to all participants, the top three were (1) not being able to meet with extended family and friends (Item 3: 69.9%); (2) worrying about the effects of COVID-19 on themselves or their family, now or in the future (Item 13: 47.5%); (3) loss of their own or family routine i.e., sleeping patterns, meal times, and/or work/school/recreational schedules (Item 5: 39.9%).

The top three COVID-19-related stresses for parents of children were (1) helping to keep their child a safe distance from extended family, and preventing them from visiting extended family i.e., grandparents (Item 16: 11.9%); (2) their child’s school closing (Item 14: 9.9%); and helping their child avoid crowded places and activities they like (Item 17: 8.5%).

The top three COVID-19-related stresses for people with older, aging parents were (1) worrying that their aging parents would become infected with COVID-19 (Item 24: 44.7%); (2) worrying that aging parents will not receive adequate medical care if they become infected with COVID-19 (Item 25: 36.9%); and worrying that their aging parents will become lonely during the COVID-19 crisis (Item 22: 34.1%).

The top three COVID-19-related feelings of gratitude were personal and familial health (Item 26: 85.4%), relationships with extended family and friends (Item 27: 81.7%), and their job (Item 28: 61.9%). Participants with aging parents were most grateful for their aging parents’ health and safety (Item 34; 67.9%). Participants with children were most grateful for their child’s relationships with their friends (Item 32: 21.7%).

### 3.6. What Were the Psychometric Properties of the ECQ?

Each a priori ECQ scale had satisfactory internal consistency reliability: Personal Stress, items 1–13, alpha = 0.79; Parenting Stress, items 14–21, alpha = 0.90; Older Parent Stress, items 22–25 alpha = 0.80; and Gratitude, items 26–34, alpha = 0.72.

An exploratory factor analysis of ECQ items yielded factors that corresponded to a priori subscales with one exception. Items in the Personal Stress subscale loaded on two separate factors that assessed “Routines and Resources” (items 2, 3, 5, 6, 7, and 13; alpha = 0.61) and “Worry and Well-being” (items 8–12; alpha = 0.87). Since the alpha value for the 13-item Personal Distress scale was satisfactory (0.79), this scale rather than the two subfactors was used in the main analysis.

A five-band severity classification was devised based on the categorization of the DASS-21, which the ECQ was compared to. Within the ECQ subscales, the following ranges were derived through k-means clustering for Personal Distress: Normal 0–12; Mild 13–2; Moderate 20–26; Severe 27–33; Extremely Severe >34; Older Aging Parents: Normal 0–4; Mild 5–2; Moderate 8–11; Severe 12–15; Extremely Severe >16; Parents to children: Normal 0–8; Mild 9–2; Moderate 16–21; Severe 22–29; Extremely Severe >30.

### 3.7. Were the COVID-19 Related Stresses and Gratitude Assessed by the ECQ Correlated with Indices of Mental Health and Well-Being?

Table 5 shows correlations between the ECQ and other scales. The ECQ Personal Stress, Parenting Stress, and Older Parent Stress scales had significant (*p* < 0.001) correlations with DASS-21 Depression, Anxiety, and Stress scales completed to reflect distress during quarantine, the WEMWBS well-being scale, and the BIPQ Perception of COVID-19 and Emotional Impact of COVID-19 scales. All correlations were in the expected direction. High levels of COVID-19-related stresses assessed with the ECQ were associated with greater depression, anxiety, and stress assessed with the DASS-21; lower levels of well-being assessed with the WEMWBS; and greater negative perceptions of and emotional reactions to COVID-19 assessed with the BIPQ. The ECQ Gratitude scale had non-significant correlations with the DASS-21 Depression, Anxiety and Stress Scale when stratified by group. The ECQ Gratitude scale correlated positively and significantly with the WEMWBS for people with older aging parents and individuals with neither older aging parents nor children. There was a positive, non-significant correlation for parents of children. There were also positive and significant correlations between the ECQ Gratitude measure and the BIPQ Perception of COVID-19 for all group stratifications. The Emotional Impact of COVID-19, as measured by the BIPQ, correlated positively and significantly with the ECQ Gratitude scale for parents and people with older parents. The BIPQ Knowledge about COVID-19 had a significant correlation with the ECQ Gratitude scale for parents of children. Supplementary Tables S3 and S4 report the correlation coefficients across all measures, stratified by group.

## 4. Discussion

This study utilized a cross-sectional online survey-based approach to investigate stress, anxiety, depression, and psychological well-being with members of the Irish public recruited through national media and social media outlets. We investigated changes in psychological distress during the COVID-19 pandemic comparing psychological outcomes before and during the quarantine period in Ireland with a sample of individuals ranging age from 18 to 76 years. Participants were asked to retrospectively comment on their “before lockdown” experience of mood and well-being. We addressed a series of five research questions. With regard to the first question concerning mean levels and rates of depression, anxiety, and stress, we found that both mean levels of all three variables and clinical levels of symptoms increased significantly during the quarantine period. The greatest increase in case severity occurred for depression. This is in line with current research specific to the COVID-19 pandemic [10]. With regard to the second question concerning mean reports of depression, anxiety, and stress in subgroups of the sample, we found that increases in depression, anxiety and stress from pre-quarantine to quarantine were not significantly affected by having responsibility for caring for a child or older, aging parents. With regard to the third question concerning rates of stresses and things for which people felt grateful that were specifically related to the COVID-19 crisis, the most frequently identified stressors by the whole sample related to social isolation, personal and familial well-being, and loss of routines such as sleeping patterns and recreational schedules. For those caring for children, the most frequently identified stressors related to keeping their child safe, keeping their child away from crowded areas, and their child’s school closing, which was in line with expected outcomes [25]. For those with older, aging parents, the most frequently identified stress was them contracting COVID-19. There was also concern over the availability of medical treatment for their parent should they need it, followed by stress that their older aging parent may become lonely during the pandemic. The most frequently identified thing that people indicated the COVID-19 crisis made them grateful for was their own personal and familial health, close relationships, and their current employment.

The fourth question concerned the psychometric properties of the ECQ. We found that the four a priori subscales had acceptable levels of internal consistency reliability, and that the structure of the ECQ was partially supported by factor analytic results. We also created severity classification bands for the ECQ based on the DASS-21, which has been used to identify high levels of psychological distress during the COVID-19 pandemic through a national survey in Italy [10]. The final question concerned the association between COVID-19-related stresses and gratitude assessed by the ECQ and indices of mental health and well-being. We found that three of the four ECQ scales had significant correlations in expected directions with depression, anxiety, stress, and each correlated as expected with mental health well-being and perceptions of COVID-19.

A limitation of the current study is the profiling of parents, children, and older aging parents. While our public sample allows for stratification into these groups to better understand relative distress, our questionnaire was not designed to specifically investigate specific additional stressors, i.e., parents who may be managing behaviors that challenge during the quarantine period, or caregiving demands for older aging parents. It is well reported in the literature that externalizing behavior in children is among the most prevalent causes of parental stress across a number of clinical morbidities [32], and quarantine, social isolation, school closure, and reduced access to routine coping strategies may result in greater parental stress. However, such a conclusion cannot be drawn from this study [25,26]. A further limitation is the lack of male participants (16.4%), which future research could aim to recruit specifically, as this may implicate the generalizability of the findings. Lastly, the cross-sectional nature of the study, which included a retrospective component, may be subject to memory bias, and thusly participants may have reported lower scores on measures of distress prior to quarantine. Future research could prospectively conduct serial assessments. Our study is in line with the existing literature on psychological outcomes in response to both COVID-19 and what we know of the psychological impact of quarantine [11,23,27,33].

## 5. Conclusions

The Irish general public requires increased access to mental health services to meet the increase in COVID-19-related psychological problems identified in this study. In particular, access to evidence-based psychological interventions are needed that support the development and maintenance of coping mechanisms, in order to improve mental health and psychological well-being. Specifically, this research highlights the need to support people and their families across the lifespan (18–76 years) within our healthcare systems, with both traditional and non-traditional intervention strategies, ensuring accessibility is considered. This research suggests that features of depression have significantly increased as a result of the COVID-19 pandemic, which are further perpetuated by the quarantine and imposed restriction, albeit necessary steps for infection control.

## Figures and Tables

**Table 1:** Demographics of the total group stratified by group membership.

			Total Cohort (*n* = 847)	Parent to a Child (*n* = 269) *	Older Aging Parent (*n* = 433)	Neither (*n* = 145)
Gender	Female	%	83.6	83	84.8	80
Age	(Range:18–76)	M ± SD	36.07 ± 10.29	41.07 ± 6.95	33.26 ± 8.61	35.19 ± 15.49
Household income	0–24	%	9	6	9.7	13.1
	25–49		26	16.4	28.9	35.2
	50–74		24.4	24.9	25.6	20.0
	75–99		19.1	23.8	17.1	16.6
	100–149		15.1	18.6	13.9	12.4
	≥150 k		6	10.4	4.4	2.8
Education	≤School	%	22.5	23.2	16.7	24.8
	Degree		38.7	34.9	41.9	36.6
	Masters		32.5	27.9	35	33.8
	Doctorate		5.28	5.6	5.3	4.1
	Post-doc		0.8	0.4	1.2	0.7
Relationship Status	Single	%	7.6	3	8.3	13.1
	Engaged		10.5	5.6	11.8	14.5
	Married		43	76.2	27.7	22.8
	Committed		38.9	3.8	50.1	48.3
Currently Working?	Yes	%	64.4	64.3	68.6	51.7

Note: “Neither” refers to neither a child nor an older aging parent; * Of which, *n* = 33 only have a child as illustrated in Figure 1.

**Table 2:** DASS-21 depression, anxiety, and stress mean scores before and during quarantine.

Variable		Total Sample	Parent to a Child	Older Aging Parent	Neither
*n*		847	269	433	145
**Depression**	Pre-Q	7.46 ± 7.78	6.59 ± 6.81	8.12 ± 8.04	7.09 ± 8.54
	Q	10.54 ± 9.46	9.14 ± 8.74	11.41 ± 7.12	10.51 ± 9.89
**Anxiety**	Pre-Q	5.39 ± 5.87	4.71 ±5.71	5.82 ± 5.88	5.34 ± 6.04
	Q	6.02 ± 6.86	5.29 ± 6.67	6.63 ± 7.12	5.56 ± 6.26
**Stress**	Pre-Q	11.99 ± 7.74	11.41 ± 7.40	12.46 ± 8.00	11.62 ± 7.49
	Q	12.86 ± 9.09	11.73 ± 8.63	13.45 ± 9.24	13.20 ± 9.33

**Note:** DASS-21 = Depression, Anxiety, and Stress Scale. Pre-Q = Pre-quarantine. Q = During Quarantine. Standard deviations are given after ±. For each variable, means with different superscripts differ significantly at *p* < 0.01.

**Table 3:** A summary of the total percentage of the cohort who score within the cut-off categories of the DASS-21 for Stress, Anxiety, and Depression, stratified by group membership.

			Total Cohort (*n* = 847)				Parents of Children (*n* = 269) *				Older Aging Parents (*n* = 433)				Neither Children nor Older Aging Parent (*n* = 145)			
Variable	DASS-21 Classification	Range	Before ^1^ (%)	Mean ± SD ^2^	During ^3^ (%)	Mean ± SD ^2^	Before (%)	Mean ± SD	During (%)	Mean ± SD	Before (%)	Mean ± SD	During (%)	Mean ± SD	Before (%)	Mean ±SD	During (%)	Mean ± SD
Stress	Normal	0–14	72.3	8.22 ± 4.21	66	7.62 ± 4.69	76.4	8.24 ± 4.34	71.3	7.32 ± 4.77	69.7	8.27 ± 4.15	64	7.93 ± 4.60	72.9	4.01 ± 2.09	62.3	3.63 ± 2.38
	Mild	15–18	11.5	16.92 ± 1.01	12.3	16.90 ± 1.01	9.7	17.04 ± 1.01	9.8	16.56 ± 0.916	12.6	16.90 ± 1.00	13.4	16.98 ± 1.00	11.8	8.41 ± 0.507	13.8	8.57 ± 0.507
	Moderate	19–25	10.1	21.78 ± 1.53	10.8	22.04 ± 1.58	9.3	21.50 ± 1.21	9.4	22.08 ± 1.50	10.4	21.86 ± 1.69	10.9	22.00 ± 1.59	10.4	11.00 ± 0.755	13	11.05 ± 0.872
	Severe	26–33	4	28.36 ± 1.96	7.7	28.38 ± 2.04	3.5	28.88 ± 1.45	7.9	28.10 ± 2.10	4.5	28.10 ± 2.05	8	28.42 ± 2.16	3.5	14.20 ± 1.30	6.5	14.44 ± 0.726
	Extremely Severe	≥34	2.1	36.94 ± 3.17	3.1	37.68 ± 3.24	1.2	39.55 ± 3.05	1.6	37.00 ± 3.82	2.8	36.33 ± 3.28	3.6	38.00 ± 3.09	1.4	18.50 ± 0.707	4.3	17.66 ± 1.03
Anxiety	Normal	0–7	69.3	2.22 ± 2.16	67.5	2.26 ± 2.17	73.8	1.95 ± 2.19	72.8	1.97 ± 2.16	66.4	2.14 ± 2.12	63.9	2.53 ± 2.17	70	2.04 ± 2.16	68.3	2.06 ± 2.13
	Mild	8–9	8.9	8.00 ± 0.00	8.2	8.00 ± 0.00	7.4	8.00 ± 0.00	7	8.00 ± 0.00	10.9	8.00 ± 0.00	9.2	8.00 ± 0.00	5.7	8.00 ± 0.00	7.2	8.00 ± 0.00
	Moderate	10–14	14.8	11.45 ± 1.61	14.8	11.53 ± 1.54	13.3	11.52 ± 1.63	11.7	11.66 ± 1.39	15.4	11.47 ± 1.63	16.2	11.37 ± 1.56	15.7	11.27 ± 1.57	16.5	11.82 ± 1.69
	Severe	15–19	2.6	16.38 ± 0.80	3.7	17.20 ± 0.99	2.3	16.00 ± 0.00	1.9	17.60 ± 0.894	2.4	16.60 ± 0.996	4.6	17.15 ± 1.01	3.6	16.40 ± 0.894	4.3	17.00 ± 1.09
	Extremely Severe	≥20	4.4	23.27 ± 3.82	5.8	25.82 ± 5.48	3.1	24.75 ± 4.65	6.6	24.11 ± 4.27	5	23.14 ± 3.26	6.1	27.20 ± 5.85	5	22.00 ± 4.47	3.6	24.80 ± 6.41
Depression	Normal	0–9	70	3.36 ± 2.66	53.7	3.64 ± 2.75	74	3.28 ± 2.65	60.5	3.41 ± 2.77	66.1	3.52 ± 2.65	49.4	3.89 ± 2.70	74.5	3.10 ± 2.70	54	3.46 ± 2.85
	Mild	10–13	11.5	10.88 ± 0.99	15.1	11.20 ± 0.982	9.5	11.04 ± 1.01	12.6	11.06 ± 1.01	13.2	10.75 ± 0.977	15.8	11.23 ± 0.980	9.9	11.14 ± 1.02	17.3	11.33 ± 0.963
	Moderate	14–20	11.7	16.43 ± 2.29	17.1	16.43 ± 2.19	12.6	16.24 ± 2.22	15.4	16.30 ± 2.22	12	16.31 ± 2.37	19.5	16.55 ± 2.15	9.2	17.38 ± 2.06	12.9	16.22 ± 2.36
	Severe	21–27	3.5	24.06 ± 1.64	7.0	23.78 ± 1.60	2.3	23.33 ± 1.63	5.5	23.14 ± 1.51	4.9	24.19 ± 1.66	7.1	23.93 ± 1.64	1.4	25.00 ± 1.41	9.4	24.15 ± 1.51
	Extremely Severe	≥28	3.3	33.40 ± 4.76	7.2	33.72 ± 4.99	1.5	34.50 ± 5.74	5.9	31.86 ± 4.03	3.8	32.62 ± 4.36	8.3	33.94 ± 5.24	5	34.57 ± 5.50	6.5	36.00 ± 4.79

**Note:**^1^ “Before” refers to the time period before the government-enforced quarantine restrictions; ^2^ The mean and standard deviation of the group performance within the DASS-21 Classification, stratified into Stress, Anxiety, and Depression subscales; ^3^ “During” refers to the time period during the quarantine. * Of which, *n* = 33 only have a child, as illustrated in Figure 1.

**Table 4:** COVID-19-related sources of stress and feelings of gratitude.

Item No.	COVID-19-Related Personal Stress for all Participants (*n* = 847)	%
	**In the past month, how much stress have you experienced as a result of the following things?**	“Quite a lot” or “A great deal”
3	Not being able to meet with your extended family and friends	69.9
13	Worrying about the effects COVID-19 on you or your family, now or in the future	47.5
5	Loss of your own or your family’s daily routine (such as sleeping patterns; meal times; work, school and recreation schedules)	39.9
9	Worrying that you may become infected with COVID-19 and then infect other people	35.8
10	You, or members of your family being hospitalized for COVID-19 illness	26.4
11	Death of a family member or very close friend as a result of COVID-19	23.3
12	Witnessing others in your community suffering because of COVID-19	20.3
7	Getting a lot of conflicting information and misinformation online and in the media about COVID-19	17.9
8	You or members of your family becoming ill with COVID-19	16.3
1	Financial hardship for you or your family arising from the COVID-19 crisis due to job loss or loss of earnings	15.7
2	Having difficulty getting supplies when you need them, including face masks, hand sanitizers, medicines, food, drinks, or other essentials	12.6
6	Family conflict arising from the COVID-19 crisis due to arguing or fighting with other family members more than usual because you are spending more time together at home	9
4	Not being able to go to your church or place of religious worship	4.9
**COVID-19-related stresses for parents to a child (*n* = 268)**		
16	Helping your child keep a safe distance from members of your extended family or preventing them from visiting with the extended family (for example grandparents)	11.9
14	Your child’s school closing	9.9
17	Helping your child avoid crowded places and activities that they like, such as going to sports or musical events, scouts or guides, clubs, the playground, or to church	8.5
15	Helping your child keep a safe distance from their friends or preventing them from mixing with their friends	8.1
18	Helping your child to not shake hands, hug, or touch other people	5.2
19	Helping your child to wash or sanitize their hands regularly	4.8
21	Being worried that your child will catch COVID-19 because they have an underlying medical condition such as cancer or asthma, which makes them vulnerable to severe illness if they become infected	3.8
20	Helping your child remember to cough or sneeze into their elbow	2.8
**COVID-19-related stresses for people with older, aging parents (*n* = 433)**		
24	Worrying that your aging parents will become infected with COVID-19	44.7
25	Worrying that your aging parents will not receive adequate medical care if they become infected with COVID-19	36.9
22	Worrying that your aging parents will become lonely during the COVID-19 crisis	34.1
23	Worrying that your aging parents will not get supplies during the COVID-19 crisis	15.6
**COVID-19-related feelings gratitude**		
	**In the past month, how much has your experience of the COVID 19 crisis led you to feel grateful for the following things?**	
26	Your health and the health of your family (*n* = 847)	85.4
27	Your relationships with your extended family and friends (*n* = 847)	81.7
34	Your aging parents’ health and safety (*n* = 433)	67.9
28	Your job (*n* = 847)	61.9
29	Attending social, sports, and cultural events (*n* = 847)	59.7
30	Your community (*n* = 847)	45.6
32	Your child’s relationships with their friends (*n* = 268)	21.7
31	Your child’s regular attendance at school (*n* = 268)	20.7
33	Your child’s involvement in activities such as sports, music, scouts, guides, clubs, etc. (*n* = 268)	15.9

**Table 5:** Correlations between ECQ scales and DASS, WEMWBS, and BIPQ scales.

ECQ Scales						
**Scale**	**Personal Stress**	**Parenting Stress**	**Older Parent Stress**	**Gratitude Scale**		
				**Personal**	**Parents**	**Older Parent**
*n*	847	269	433	145	269	433
Quarantine DASS-21 Depression	0.39 **	0.45 **	0.24 **	0.01	−0.007	0.002
Quarantine DASS-21 Anxiety	0.46 **	0.46 **	0.32 **	0.03	0.089	0.06
Quarantine DASS-21 Stress	0.48 **	0.52 **	0.36 **	0.04	0.104	0.07
WEMWBS Well-being	−0.34 **	−0.42 **	−0.23 **	0.107 **	0.054	0.085 *
BIPQ Perception of COVID-19	0.30 **	0.21 **	0.22 **	0.183 *	0.150 *	0.240 **
BIPQ Emotional impact of COVID-19	0.53 **	0.35 **	0.41 **	0.05	0.192 **	0.178 **
BIPQ Knowledge about COVID-19	0.01	−0.02	0.03	0.031	0.161 **	0.082

Note: ECQ = Effects of COVID-19 Questionnaire. DASS-21 = Depression, Anxiety, and Stress Scale. WEMWBS = Warwick–Edinburgh Mental Well-Being Scale. BIPQ = Brief Illness Perception Questionnaire. * *p* < 0.05. ** *p* < 0.001.

## Supplementary Materials

The following are available online at https://www.mdpi.com/2077-0383/9/11/3481/s1, Table S1: Effects of COVID-19 questionnaire (ECQ); Table S2: Mean and standard deviation of the psychological outcome measures for the total cohort, and the stratified group membership; Table S3: Correlations for the psychological outcome variables for the total group and the stratification of those who had neither a child nor an older aging parent; Table S4: Correlations for the psychological outcome variables stratified by those who were parents of children, as well as participants with older aging parents.
