# Supplementary material for: Increased Psychological Distress during COVID-19 and Quarantine in Ireland: A National Survey
Source: J Clin Med. 2020 Oct 28;9(11):3481. doi: 10.3390/jcm9113481 (PMC7693396; doi:10.3390/jcm9113481)
Supplement: Supplementary file 1 [file jcm-09-03481-s001.pdf]

Table S1. Effects of COVID-19 questionnaire (ECQ).

The following questions ask about the effect that the COVID-19 or Corona virus is having on you.
For each item, click on the answer that applies to you in the **PAST MONTH.**
N/A means the item is not applicable to you.

	**In the past month, how much stress have you experienced as a result of the following things**						
1	Financial hardship for you or your family arising from the COVID-19 crisis, due to job loss, or loss of earnings	N/A 0	None 1	A little 2	Some 3	Quite a lot 4	A great deal 5
2	Having difficulty getting supplies when you need them, including face masks, hand sanitizers, medicines, food, drinks or other essentials	N/A 0	None 1	A little 2	Some 3	Quite a lot 4	A great deal 5
3	Not being able to meet with your extended family and friends	N/A 0	None 1	A little 2	Some 3	Quite a lot 4	A great deal 5
4	Not being able to go to your church or place of religious worship	N/A 0	None 1	A little 2	Some 3	Quite a lot 4	A great deal 5
5	Loss of your own, or your family's daily routine (such as sleeping patterns; meal times; work, school and recreation schedules)	N/A 0	None 1	A little 2	Some 3	Quite a lot 4	A great deal 5
6	Family conflict arising from the COVID19 crisis, due to arguing, or fighting with other family members more than usual because you are spending more time together at home	N/A 0	None 1	A little 2	Some 3	Quite a lot 4	A great deal 5
7	Getting a lot of conflicting information and misinformation online and in the media about COVID-19	N/A 0	None 1	A little 2	Some 3	Quite a lot 4	A great deal 5
8	You, or members of your family becoming ill with COVID-19	N/A 0	None 1	A little 2	Some 3	Quite a lot 4	A great deal 5
9	Worrying that you may become infected with COVID-19 and then infect other people	N/A 0	None 1	A little 2	Some 3	Quite a lot 4	A great deal 5
10	You, or members of your family being hospitalised for COVID-19 illness	N/A 0	None 1	A little 2	Some 3	Quite a lot 4	A great deal 5
11	Death of a family member or very close friend as a result of COVID-19	N/A 0	None 1	A little 2	Some 3	Quite a lot 4	A great deal 5
12	Witnessing others in your community suffering because of COVID-19	N/A 0	None 1	A little 2	Some 3	Quite a lot 4	A great deal 5
13	Worrying about the effects COVID-19 on you or your family, now or in the future	N/A 0	None 1	A little 2	Some 3	Quite a lot 4	A great deal 5
	**If you have children**						
14	Your child's school closing	N/A 0	None 1	A little 2	Some 3	Quite a lot 4	A great deal 5

15	Helping your child keep a safe distance from their friends, or preventing them from mixing with their friends	N/A 0	None 1	A little 2	Some 3	Quite a lot 4	A great deal 5
16	Helping your child keep a safe distance from members of your extended family, or preventing them from visiting with the extended family (for example grandparents)	N/A 0	None 1	A little 2	Some 3	Quite a lot 4	A great deal 5
17	Helping your child avoid crowded places, and activities that they like, such as going to sports or musical events, scouts or guides, clubs, the playground, or to church	N/A 0	None 1	A little 2	Some 3	Quite a lot 4	A great deal 5
18	Helping your child to not shake hands, hug, or touch other people	N/A 0	None 1	A little 2	Some 3	Quite a lot 4	A great deal 5
19	Helping your child to wash or sanitise their hands regularly	N/A 0	None 1	A little 2	Some 3	Quite a lot 4	A great deal 5
20	Helping your child to remember to cough or sneeze into their elbow	N/A 0	None 1	A little 2	Some 3	Quite a lot 4	A great deal 5
21	Being worried that your child will catch COVID-19 because they have an underlying medical condition such as cancer or asthma, that makes them vulnerable to severe illness if they become infected	N/A 0	None 1	A little 2	Some 3	Quite a lot 4	A great deal 5
	**If you have aging parents**						
22	Worrying that your aging parents will become lonely during the COVID-19 crisis	N/A 0	None 1	A little 2	Some 3	Quite a lot 4	A great deal 5
23	Worrying that you aging parents will not get supplies during the COVID-19 crisis	N/A 0	None 1	A little 2	Some 3	Quite a lot 4	A great deal 5
24	Worrying that your aging parents will become infected with COVID-19	N/A 0	None 1	A little 2	Some 3	Quite a lot 4	A great deal 5
25	Worrying that your aging parents will not receive adequate medical care if they become infected with COVID-19	N/A 0	None 1	A little 2	Some 3	Quite a lot 4	A great deal 5
	**In the past month, how much has your experience of the COVID 19 crisis led you to feel grateful for the following things**						
26	Your health, and the health of your family	N/A 0	None 1	A little 2	Some 3	Quite a lot 4	A great deal 5
27	Your relationships with your extended family and friends	N/A 0	None 1	A little 2	Some 3	Quite a lot 4	A great deal 5
28	Your job	N/A 0	None 1	A little 2	Some 3	Quite a lot 4	A great deal 5
29	Attending social, sports, and cultural events	N/A 0	None 1	A little 2	Some 3	Quite a lot 4	A great deal 5
30	Your community	N/A 0	None 1	A little 2	Some 3	Quite a lot 4	A great deal 5

31	Your child’s regular attendance at school	N/A<br>0	None<br>1	A<br>little<br>2	Some<br>3	Quite<br>a lot<br>4	A<br>great<br>deal<br>5
32	Your child’s relationships with their friends	N/A<br>0	None<br>1	A<br>little<br>2	Some<br>3	Quite<br>a lot<br>4	A<br>great<br>deal<br>5
33	Your child’s involvement in activities such as sports, music, scouts, guides, clubs etc	N/A<br>0	None<br>1	A<br>little<br>2	Some<br>3	Quite<br>a lot<br>4	A<br>great<br>deal<br>5
34	Your aging parents health and safety	N/A<br>0	None<br>1	A<br>little<br>2	Some<br>3	Quite<br>a lot<br>4	A<br>great<br>deal<br>5

Note: Items 1–13 COVID-19 personal stress scale. Items 14–21 COVID-19 parenting stress scale. Items 22–25 COVID-19 aging parents stress scale. Items 26–33 COVID-19 gratitude scale. To get a scale score, sum scores of items in the scale and divide this by the number of items which did not have an N/A response.

Table S2. Mean and Standard Deviation of the Psychological Outcome Measures for the total cohort, and the stratified group membership.

		Total Cohort (*n* = 847)	Parent to a child (*n* = 269)*	Older aging parent (*n* = 433)	Neither (*n* = 145)
Psychological Outcomes	BIPQ Cognitive	29.94 ± 5.21	29.85 ± 5.31	30.07 ± 5.22	29.76 ± 5.05
	DASS Stress	11.99 ± 7.74	11.41 ± 7.40	12.46 ± 8.00	11.62 ± 7.49
	DASS Anxiety	5.39 ± 5.87	4.71 ± 5.71	5.82 ± 5.88	5.34 ± 6.04
	DASS Depression	7.46 ± 7.78	6.59 ± 6.81	8.12 ± 8.04	7.09 ± 8.54
	(Quarantine) DASS Stress	12.86 ± 9.09	11.73 ± 8.63	13.45 ± 9.24	13.20 ± 9.33
	(Quarantine) DASS Anxiety	6.02 ± 6.86	5.29 ± 6.67	6.63 ± 7.12	5.56 ± 6.26
	(Quarantine) DASS Depression	10.54 ± 9.46	9.14 ± 8.74	11.41 ± 7.12	10.51 ± 9.89
	WEMWBS	53.98 ± 10.14	54.16 ± 10.06	53.91 ± 9.91	53.86 ± 11.01
	ECQ—Personal	19.41 ± 7.85	20.25 ± 7.74	19.14 ± 7.85	18.63 ± 7.96
	ECQ—Parent	-	10.43 ± 6.74	-	-
	ECQ—OAP	6.47 ± 4.91	7.09 ± 4.61	8.20 ± 4.11	-
	ECQ—Gratitude	18.84 ± 6.42	23.85 ± 6.71	17.44 ± 4.35	13.56 ±4.72
	BIPQ—Knowledge	7.80 ± 1.76	7.88 ± 1.87	7.75 ± 1.74	7.76 ± 1.59
	BIPQ—Emotional	6.12 ± 2.29	6.03 ± 2.34	6.15 ± 2.24	6.21 ± 2.32

ECQ: Effects of COVID-19 Questionnaire; OAP; Older Aging Parent; WEMWBS: Warwick-Edinburgh Mental Wellbeing Scale; BIPQ: Brief Illness Perception Questionnaire; DASS: Depression Anxiety Stress Scale; **Note:** Quarantine refers to the period of time where participants were invited to complete the DASS-21 during the period of government-imposed restrictions; 'Neither' refers to neither a child nor an older aging parent. *Of which, *n*=33 only have a child as illustrated in Figure 1.

Table S3. Correlations for the psychological outcome variables for the Total Group and the stratification of those who had neither a child nor an older aging parent.

	Variables		1	2	3	4	5	6	7	8	9	10	11	12	13	14		
1	ECQ	*r*	ECQ	-	-	.200*	-268*	.288**	.285**	.315**	.367**	.368**	.341**	.377**	-.103	.446**	1	ECQ
	Personal	*p*	Personal	-	-	.027	.003	.002	.002	<.001	<.001	<.001	<.001	<.001	.262	<.001		Personal
2	ECQ Parent	*r*	-	ECQ	-	-	-	-	-	-	-	-	-	-	-	-	2	ECQ Parent
		p	-	Parent	-	-	-	-	-	-	-	-	-	-	-	-		
3	ECQ OAP	*r*	.411**	-	ECQ	-	-	-	-	-	-	-	-	-	-	-	3	ECQ OAP
	Distress	*p*	<.001	-	OAP	-	-	-	-	-	-	-	-	-	-	-		Distress
4	ECQ	*r*	.247**	-	.312**	ECQ	.239**	.162	-.053	.016	.007	.024	.005	.028	-.017	.044	4	ECQ
	Grateful	*p*	<.001	-	<.001	Grateful	.008	.076	.567	.861	.940	.794	.959	.757	.857	.634		Grateful
5	WEMWBS	*r*	-.342**	-	-	.048	WEM-	.080	-.545**	-	-.333**	-.581**	-.453**	-.394**	.128	-.382**	5	WEMWBS
		p	<.001	-	.144**	.181	WBS	.382	<.001	.391**	<.001	<.001	<.001	<.001	.161	<.001		
					<.001					<.001								
6	BIPQ	*r*	.296**	-	.127**	.138**	-.064	BIPQ	-.105	-.125	-.129	.079	.170	.087	.253**	.387**	6	BIPQ
	Cognitive	*p*	<.001	-	<.001	<.001	.078	Cognitive	.251	.172	.158	.387	.062	.341	.005	<.001		Cognitive
7	DASS	*r*	.202**	-	.111**	-.044	-.372**	-.002	DASS	.752**	.626**	.678**	.502**	.484**	-.217*	.354**	7	DASS
	Depression	*p*	<.001	-	.002	.226	<.001	.957	Depression	<.001	<.001	<.001	<.001	<.001	.017	<.001		Depression
8	DASS	*r*	.254**	-	.142**	.036	-.249**	.049	.691**	DASS	.731**	.461**	.567**	.509**	-.146	.262**	8	DASS
	Stress	*p*	<.001	-	<.001	.312	<.001	.172	<.001	Stress	<.001	<.001	<.001	<.001	.109	.004		Stress
9	DASS	*r*	.274**	-	.149**	-.011	-.228**	.023	.590**	.681**	DASS	.424**	.488**	.719**	-179*	.216*	9	DASS
	Anxiety	*p*	<.001	-	<.001	.763	<.001	.519	<.001	<.001	Anxiety	<.001	<.001	<.001	.049	.017		Anxiety
10	Quarantine	*r*	.391**	-	.183**	-.011	-.631**	.125**	.552**	.389**	.379**	DASS	.780**	.650**	-.187*	.525**	10	Quarantine
	Depression	*p*	<.001	-	<.001	.754	<.001	.001	<.001	<.001	<.001	Depression	<.001	<.001	.040	<.001		Depression
												Quarantine						
11	Quarantine	*r*	.476**	-	.244**	.037	-.542**	.158**	.405**	.513**	.420**	.731**	DASS	.727**	-.183*	.507**	11	Quarantine
	Stress	*p*	<.001	-	<.001	.306	<.001	<.001	<.001	<.001	<.001	<.001	Stress	<.001	.044	<.001		Stress

Neither Child nor OAP

			1	2	3	4	5	6	7	8	9	10	11	12	13	14		
													Quarantine					
12	Quarantine	*r*	.458**	-	.247**	.044	-.417**	.116**	.383**	.410**	.630**	.649**	.717**	DASS	-.140	.410**	12	Quarantine
	Anxiety	*p*	<.001	-	<.001	.222	<.001	.001	<.001	<.001	<.001	<.001	<.001	Anxiety	.127	<.001		Anxiety
														Quarantine				
13	Understand	*r*	.005	-	-.016	.077*	.123**	.283**	-.116**	-.059	-.017	-.105**	-.062	-.011	BIPQ	.029	13	Understand
	COVID	*p*	.884	-	.667	.032	.001	<.001	.001	.099	.632	.004	.086	.752	COVID	.752		COVID
14	Emotional	*r*	531**	-	.220**	.133**	-.457**	.392**	.248**	.294**	.286**	.485**	.501**	.466**	.082*	BIPQ	14	Emotional
	Impact	*p*	<.001	-	<.001	.002	<.001	<.001	<.001	<.001	<.001	<.001	<.001	<.001	.023	Emotional		Impact

Shading: shaded upper segment reflects participants with neither a child nor an aging parent (listwise, n=121)**;** lower quadrant reflects total cohort (listwise n=769). **Abbreviations:** ECQ: Effects of COVID-19 Questionnaire; OAP; Older Aging Parent; WEMWBS: Warwick-Edinburgh Mental Wellbeing Scale; BIPQ: Brief Illness Perception Questionnaire; DASS: Depression Anxiety Stress Scale; r: correlation coefficient; p: significance value; **Note:** Quarantine refers to the period of time where participants were invited to complete the DASS-21 during the period of government-imposed restrictions; * p<0.05; **p<0.001;.

Table S4. Correlations for the psychological outcome variables stratified by those who were parents of children, as well as participants with older aging parents.

	Variables		1	2	3	4	5	6	7	8	9	10	11	12	13	14		
1	ECQ	*r*	ECQ	-	.600**	.258**	-.365**	.315**	.210**	.256**	.257**	.390**	.488*	.434**	.032	.583**	1	ECQ
	Personal	*p*	Personal	-	<.001	<.001	<.001	<.001	<.001	<.001	<.001	<.001	<.001	<.001	.524	<.001		Personal
2	ECQ Parent	*r*	.506**	ECQ	-	-	-	-	-	-	-	-	-	-	-	-	2	ECQ Parent
		p	<.001	Parent	-	-	-	-	-	-	-	-	-	-	-	-		
3	ECQ OAP	*r*	.433**	.308**	ECQ	.261**	-.232**	.217**	.139**	1.58**	.205**	.244**	.359**	.321**	.029	.409**	3	ECQ OAP
	Distress	*p*	<.001	<.001	OAP	<.001	<.001	<.001	.005	.002	<.001	<.001	<.001	<.001	.563	<.001		Distress
4	ECQ	*r*	.285**	.328**	.201**	ECQ	.075	.244**	-.031	.061	.045	.003	.077	.064	.059	.167**	4	ECQ
	Grateful	*p*	<.001	<.001	.001	Grateful	.133	<.001	.542	.221	.366	.946	.123	.199	.238	.001		Grateful
5	WEMWBS	*r*	-.346**	-.415**	-.140*	-.039	WEM-	-.115*	-.332**	-	-.204**	-.659**	-.594**	-.465**	.134**	-.495**	5	WEMWBS
		p	<.001	<.001	.027	.540	WBS	.021	<.001	.232**	<.001	<.001	<.001	<.001	.008	<.001		
										<.001								

Parents of Children$

Participants with OAP

6	BIPQ	*r*	.281**	.205**	.149*	.158*	-.044	BIPQ	.038	.101*	.086	.147**	.127*	.092	.253**	.376**	6	BIPQ
	Cognitive	*p*	<.001	.001	.019	.012	.490	Cognitive	.452	.044	.085	.003	.011	.065	<.001	<.001		Cognitive
7	DASS	*r*	.154*	.196**	.101	-.015	-.346**	-.023	DASS	.698**	.584**	.501**	.367**	.358**	-.090	.218**	7	DASS
	Depression	*p*	.015	.002	.111	.818	<.001	.717	Depression	<.001	<.001	<.001	<.001	<.001	.071	<.001		Depression
8	DASS	*r*	.237**	.250**	.185**	.117	-.210**	.035	.639**	DASS	.681**	.365**	.473**	.364**	-.036	.280**	8	DASS Stress
	Stress	*p*	<.001	<.001	.003	.066	.001	.581	<.001	Stress	<.001	<.001	<.001	<.001	.468	<.001		
9	DASS	*r*	.286**	.254**	.177**	.077	-.214**	-.019	.573**	.650**	DASS	.365**	.383**	.578**	-.006	.265**	9	DASS
	Anxiety	*p*	<.001	<.001	.005	.227	.001	.767	<.001	<.001	Anxiety	<.001	<.001	<.001	.913	<.001		Anxiety
10	Quarantine	*r*	.442**	.445**	.227**	.109	-.616**	.106	.567**	.384**	.354**	DASS	.737**	.686**	-.101*	.495**	10	Quarantine
	Depression	*p*	<.001	<.001	<.001	.086	<.001	.095	<.001	<.001	<.001	Depression	<.001	<.001	.043	<.001		Depression
												Quarantine						
11	Quarantine	*r*	.552**	.520**	.280**	.164**	-.500**	.203**	.415**	.553**	.433**	.683**	DASS	.720**	-.099*	.493**	11	Quarantine
	Stress	*p*	<.001	<.001	<.001	.009	<.001	.001	<.001	<.001	<.001	<.001	Stress	<.001	.048	<.001		Stress
													Quarantine					
12	Quarantine	*r*	.562**	.456**	.274**	.163**	-.349**	.166**	.369**	.438**	.675**	.568**	.704**	DASS	-.028	.459**	12	Quarantine
	Anxiety	*p*	<.001	<.001	<.001	.010	<.001	.009	<.001	<.001	<.001	<.001	<.001	Anxiety	.584	<.001		Anxiety
														Quarantine				
13	Understand	*r*	.005	-.019	-.060	.137*	.107	.335**	-.108	-.056	.042	-.070	.054	.068	BIPQ	.079	13	Understand
	COVID	*p*	.935	.754	.344	.031	.091	<.001	.088	.378	.506	.268	.397	.283	COVID	.117		COVID
14	Emotional	*r*	.504**	.353**	.186**	.211**	-.435**	.414**	.244**	.332**	.355**	.453**	.513**	.507**	.109	BIPQ	14	Emotional
	Impact	*p*	<.001	<.001	.003	.001	<.001	<.001	<.001	<.001	<.001	<.001	<.001	<.001	.086	Emotional		Impact

Shading: shaded upper segment reflects participants with only an older aging parent i.e., no children (listwise, n=399)**;** lower quadrant reflects parent cohort (listwise n=249). **Abbreviations:** ECQ: Effects of COVID-19 Questionnaire; OAP; Older Aging Parent; WEMWBS: Warwick-Edinburgh Mental Wellbeing Scale; BIPQ: Brief Illness Perception Questionnaire; DASS: Depression Anxiety Stress Scale; r: correlation coefficient; p: significance value; **Note:** Quarantine refers to the period of time where participants were invited to complete the DASS-21 during the period of government-imposed restrictions; * p<0.05; **p<0.001; §Of which, n=33 only have a child as illustrated in Figure 1.

1
